# ‘There were more wires than him’: the potential for wireless patient monitoring in neonatal intensive care

**DOI:** 10.1136/bmjinnov-2016-000145

**Published:** 2017-01-04

**Authors:** Oliver Bonner, Kathryn Beardsall, Nathan Crilly, Joan Lasenby

**Affiliations:** 1 Department of Engineering, University of Cambridge, Cambridge, UK; 2 Cambridge University Hospitals NHS Trust, Cambridge, UK

**Keywords:** neonatal intensive care, patient monitoring, wireless sensors, kangaroo care

## Abstract

**Background:**

The neonatal intensive care unit (NICU) can be one of the most stressful hospital environments. Alongside providing intensive clinical care, it is important that parents have the opportunity for regular physical contact with their babies because the neonatal period is critical for parent–child bonding. At present, monitoring technology in the NICU requires multiple wired sensors to track each baby's vital signs. This study describes the experiences that parents and nurses have with the current monitoring methods, and reports on their responses to the concept of a wireless monitoring system.

**Methods:**

Semistructured interviews were conducted with six parents, each of whom had babies on the unit, and seven nurses who cared for those babies. The interviews initially focused on the participants’ experiences of the current wired system and then on their responses to the concept of a wireless system. The transcripts were analysed using a general inductive approach to identify relevant themes.

**Results:**

Participants reported on physical and psychological barriers to parental care, the ways in which the current system obstructed the efficient delivery of clinical care and the perceived benefits and risks of a wireless system. The parents and nurses identified that the wires impeded baby–parent bonding; physically and psychologically. While a wireless system was viewed as potentially enabling greater interaction, staff and parents highlighted potential concerns, including the size, weight and battery life of any new device.

**Conclusions:**

The many wires required to safely monitor babies within the NICU creates a negative environment for parents at a critical developmental period, in terms of physical and psychological interactions. Nurses also experience challenges with the existing system, which could negatively impact the clinical care delivery. Developing a wireless system could overcome these barriers, but there remain challenges in designing a device suitable for this unique environment.

## Introduction

The neonatal intensive care unit (NICU) in a hospital attends to the needs of critically ill newborn babies, some of whom are born prematurely. The babies may weigh as little as 500 g compared with 3.5 kg, the normal birth weight of a term baby. The care provided is among the most intensive, specialised and high dependency within the hospital environment. Uniquely, the unit combines this clinical care with attending to each baby's individual developmental needs, as well as supporting the parents as they bond with their child.

**Figure 1:**
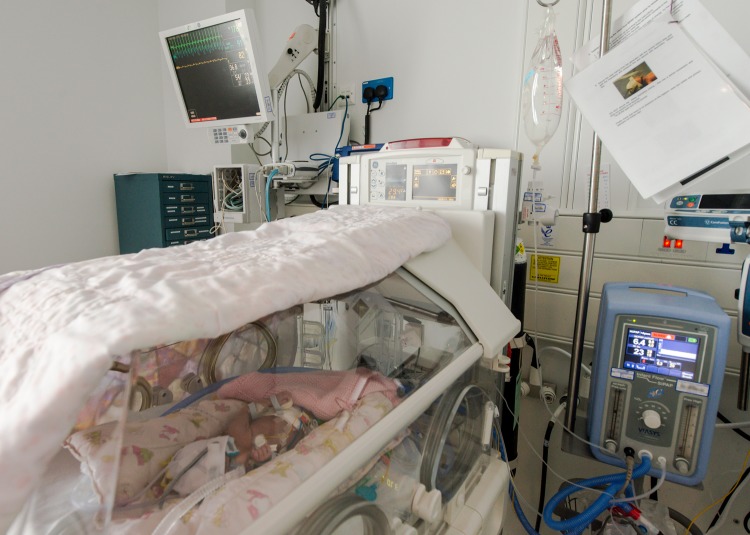
The neonatal care environment. The display in the top left visualises the vital sign signals from the ECG probes, pulse oximeter cuff and the temperature sensor. The unit in the bottom right is a non-invasive ventilator. The incubator provides a thermally (and, if required, humidity) regulated environment for the baby.

**Figure 2:**
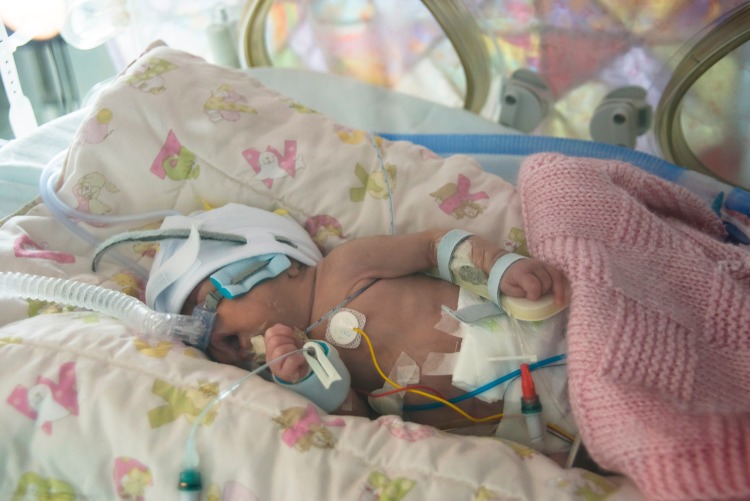
A premature baby inside an incubator in the neonatal intensive care unit. The sticker attached to the chest is one of three ECG probes. A pulse oximeter cuff is secured to the foot, and a temperature probe is attached to the baby's back. The nasal tubing provides non-invasive ventilation.

Various technologies assist the clinical teams as shown in figure 1. Babies often lie within individual incubators with some form of respiratory support via invasive or non-invasive ventilation, as well as intravenous infusion lines to administer nutrition and drugs, as shown in figure 2. In addition to these interventions, multiple monitors are used to provide continuous quantification of vital signs. The sensors used in this environment include electrocardiogram (ECG) probes (heart activity), thermistors (skin temperature) and pulse oximeters (blood oxygen saturation, SpO_2_). These sensors are all connected by wires to a monitoring unit outside of the incubator. There is also a pressure sensor connected to a separate sleep apnoea alarm. The large number of wires in and around the incubator might be reduced by introducing wireless systems, but these do not currently exist in the NICU and limited research^1^ has been conducted in implementing such a system.

Reducing the number of wires that are attached to the babies could make the delivery of clinical care easier and might encourage more physical interaction between parents and their babies. Physical interaction is an important part of parental care during the neonatal period, often taking the form of ‘kangaroo care’, where, for example, a mother places the baby against her chest for skin-to-skin contact. For those in intensive care, the babies normally would still be connected to life-supporting equipment and have limited mobility; therefore, this is always undertaken in a chair next to the incubator or cot. This practice is encouraged as part of the widely used Newborn Individualized Developmental Care and Assessment Program (NIDCAP) to enhance parent–baby bonding and has been shown to improve clinical outcomes.^2^ Kangaroo care is particularly critical for preterm neonates.^3–6^ The clinical impact of this bonding experience is pronounced, with respect to short-term physiological stability and long-term health outcomes. Benefits for the baby include less pain,^6^ better sleeping,^3^ improved weight gain^3^ ^4^ and earlier discharge.^3^ Conversely, poor parent–baby bonding ‘impairs hormonal, epigenetic and neuronal development in preterm infants’.^7^

A few studies provide a general view of how technology in the NICU affects parents and their interactions with their baby; however, there is no research exclusively focused on how sensor wires impact care in the NICU. An interview study into the effects of technology on parents in the NICU reported that they found the environment to be ‘oppressive’ and that it ‘delayed the development of their ability to participate in the care of the child’.^8^ A separate questionnaire survey identified those pieces of equipment which parents saw as a barrier to interacting with their baby^9^: the monitoring equipment was identified as a lower obstruction compared with more invasive interventions such as respiratory support or infusion lines. While these prior studies provide an important context for understanding the parents' experience of the NICU and its equipment, there are limitations. To enable the effective development of wireless solutions, we require a better understanding of how the present wired technologies are experienced and how a future wireless system might be received. The study reported here addresses this and forms part of the initial scoping exercise for the technological development of a wireless system for use in intensive care.

## Methods

### Cohort selection and recruitment

The study involved semistructured interviews^10^ with parents and nursing staff from a single NICU. The interviews were conducted at the University of Cambridge Addenbrooke's Hospital Trust in February 2016 with nurses and parents on the unit. The sample size was determined by the number of suitable parents on the unit, their emotional state and the consequent imposition on the nurses' time. Nurses were recruited into the study through distribution of information sheets; suitable parents were identified by the nurse in charge of the unit. Six parents and seven nurses were interviewed (see tables 1 and 2). For comparison, this is in line with other studies with similar participant groups.^11^ Five of the nurses were based in the NICU and two worked in the acute neonatal transfer service (ANTS), which is based at the same location. Each of the parents had one baby on the unit. Five of the babies were born at extremely low birth weight (<1 kg); three were born extremely prematurely (<28 weeks), two others very premature (28–32 weeks) and one moderately premature (32–37 weeks).^12^ ^13^

**Table 1:** Parent participants

Participant code	Time on unit
P1	4 weeks
P2	12 weeks
P3	6 weeks
P4	16 weeks
P5	6 weeks
P6	4 weeks

**Table 2:** Nurse participants

Participant code	Position
N1	Senior sister
N2	Senior sister
N3	Advanced neonatal practitioner
N4	Junior sister
N5	Senior staff nurse
N6	Junior sister (ANTS)
N7	Junior sister (ANTS)

ANTS, acute neonatal transfer service.

### Interview development

All participants were interviewed individually except the nurses from the ANTS who requested they be interviewed together. The interviews followed an established procedure for performing semistructured interviews,^10^ ^14^ and were conducted by the first author who is an electronics engineer undertaking research into developing wireless monitoring for neonates. The interview dialogue was initiated with predefined questions which were intended to elicit responses on the subject of the two main research themes:

1. current perceptions of the existing patient monitoring systems and their peripheral attachments;
2. future perceptions of a system which removes the wires between the sensor probe and the patient monitoring device.

The first part of each interview focused on eliciting the participant's views and experiences of the current systems. The second part involved introducing the concept of a wireless system and exploring the participant's views on its potential impact. The potential wireless system was described to the participants as functionally equivalent to the current setup, but with two physical changes: the connecting wires would be removed; and a small electronics module would be added to the sensor for transmitting the data to be displayed on the monitor.

Interviews with the participants were conducted in a private room near to the unit. Furthermore, a senior nurse, familiar to the parent, was present in the room to provide support or answer clinical questions. Interviews were recorded using a digital voice recorder to allow later analysis. The interviews lasted around 10 min each; a debrief was conducted post interview.

### Data analysis

Any specifically identifiable data was anonymised during interview transcription. Analysis of the interviews was performed using an established procedure for a general inductive method using thematic analysis.^14–17^ The interview transcripts were processed using ATLAS.ti (Scientific Software Development GmbH) which permits the iterative assignment of themes to segments of transcript.

## Results

The analysis led to the identification of three overall themes that are used to structure the report below, with quotations used to illustrate key points. The quotations have been edited for clarity and brevity (with edits in square brackets). Participant codes from tables 1 and 2 are included after the quotation (P*x* for parents and N*x* for nurses).

### Parental care: physical and psychological barriers

The nurses and parents discussed what they perceived to be the main obstructions to parental care in the NICU. These comprised physical and psychological barriers.

For the parents, the experience of having their baby in the NICU is clearly a very emotional one. Specific emotions that were expressed as a direct result of seeing their baby attached to the wires were intimidation, sadness, shock and fear. The parents identified their first encounter with the unit as the toughest, but as time progressed, they became more used to it*.*

Crickey. There was a lot of wires, a heck of a lot of wires—there were more wires than him. So yeah, it was quite overwhelming. [P1]

It took ages, about a month at least, [for the anxiety about the wires to settle]. [P5]

The monitoring equipment and other support systems had a significant impact on the parents' physical interaction with their babies. Most of the parents indicated a reluctance to touch their baby as a result of the wires and monitoring systems, because of the fear it induced.

It made me frightened with all the wires that were there. It made me feel very uncomfortable and I didn't sort of want to…I wanted to hold him but I felt like I couldn't because there was a lot of equipment there. [P1]

The first thing the parents always say is “look at the wires” and that's the first barrier. [N5]

The majority of the parents said that while handling their baby, they were worried about disturbing the equipment, and all of them commented that skin-to-skin contact was negatively affected by the wires (a view that was supported by the nurses). This was because of the time it took to take the baby out (and then put them back) and because the parents felt the wires were uncomfortable against the skin.

Taking the baby out for kangaroo care just takes ages. I have to detangle her, then the nurses have to take all the wires off, then they take her out, then they connect her all back up. It takes a good half an hour just to get her out for a cuddle, especially as she wriggles a lot—she does tend to tangle all her wires into one, or she pulls them out. She's a bit feisty! [P5]

Both participant groups reported that the wires cause problems for the parents and the way they interact with their baby. These problems affect their emotional state and are a barrier to physical interactions including kangaroo care.

### Delivering medical care: practical issues with the current system

The participants felt that some aspects of the existing systems presented challenges to the delivery of medical care.

The nurses generally have a positive view of the current system, as it is what they have been trained to use, and have experience of using over many years. They understand the subtle challenges that some of the systems pose—for instance the poor adherence of the ECG stickers in the humid incubators—and mitigate this by changing the ECG probes more frequently.

If you have a small baby in humidity, some of the monitoring [probes] fall off because of the humidity. So you might lose a trace of the ECG. [N2]

The temperature probe sticker was identified as adhering better, as the placement underneath the baby kept it securely attached and a stronger adhesive is used. However, one parent noted that on removal, the area of attachment could be sore. The pulse oximeter probes used on this NICU were a soft-touch, velcro-secured wrap which required changing every six hours to prevent pressure sores. One nurse commented that, owing to the fragility of the babies' skin, the probe needed to be moved frequently to avoid burns caused by heat generated from the pulse oximeter.

Achieving good contact between the sensor probe and the skin of the neonate is difficult because of the fragility of their poorly developed skin. This can lead to complications when changing the probes as the sticky pad can remove some of the baby's skin.

Very premature babies, [that is], less than 25/26 weeks, their skin is obviously more friable and they're more likely to get skin breakdown. [N1]

One of the nurses identified that excessive handling of very premature babies can have a negative impact on their development, saying that anything that would ease the transfer of the babies would greatly reduce the stress they experience. Trying to untangle wires can increase the need to move and handle the babies.

The less you can handle the very fragile babies the better. It has a massive clinical impact—stress—on very sick and premature babies. So from a developmental care point of view, even just the small differences can help, if the move from one place to another is smoother. [N6]

Half of the parents and the majority of the nurses reported that there could be a great deal of ‘clutter’ of tangled wires in the incubator. For the nurses, this caused problems when repositioning the babies; for the parents, it complicated the process of taking the babies out for kangaroo care. The ‘clutter’ is caused by the many different medical systems that are located outside of the incubator but which must be connected to the baby. Furthermore, nurses are aware of the risk of pressure sores from babies lying on wires in the incubator.

Quite often if babies have got quite a lot of monitoring on, the wires are in a bit of a knot at the bottom of the bed. [N2]

Yeah, it is awkward, having to untangle everything—untangle his leg. [P4]

One nurse highlighted baby positioning as being extremely important for their care, which she felt was hindered by the number of wires in the incubator.

Positioning is really important with premature babies—really really important, and the wires can make that [trickier]. [N6]

The participants identified these inadequacies in the current, wired system for vital sign monitoring. Wireless technologies might help to mitigate these problems if such systems were accepted in the NICU.

### Proposal for a wireless system: perceived benefits and risks

All of the participants responded positively to the idea of a wireless system when it was first introduced to them.

I think it could be a great idea actually, because if you don't have anything between the baby and the monitor, I think it's going to be easier to move the babies. [N4]

It would just be easier; I wouldn't be so scared to touch her. I think you'd be a lot more likely to touch the baby without the wires there. [P5]

Perceived benefits of the wireless system included improved comfort for the baby in the incubator because there would be no wires to lie on; a less worrying visual appearance of the baby which would reduce anxiety for the parents; and better physical interaction with the baby, in particular easier and more comfortable kangaroo care.

I think it would be great to have it be wireless, if it can be as effective [as the present system]. As a parent you become a little bit obsessed by them [the wires] at first, because they take your attention away from looking [at your baby]. Certainly [having a wireless system] would improve things, [it would] make you feel more comfortable. You'd be less entangled. [P3]

It would be really good when we take babies out for kangaroo care, obviously all these wires trailing about causes a lot of problems. Especially if there are lots of fluids lines as well. So that would make a huge difference. [N1]

Three of the nurses (including the two nurses from ANTS) indicated that a wireless system would be beneficial when babies are being transported, whether within or between hospitals. The simplification of the movement process was identified as the main reason that a wireless system would help in this setting.

Well I think it would be great on transport. We have very limited space as it is with the incubators—they're very small, very compact. So having a baby where you haven't got all the additional wires and all the cabling coming in [would be] a plus point. [N7]

Some of the participants raised concerns regarding a wireless system, including issues related to battery life, reliability of the wireless link, ensuring the right sensor connects to the right monitor and quality of data not being compromised. Another concern was heating of the babies from the sensor unit, lest it damage their extremely fragile skin. One nurse was worried that without obvious wires to act as a reminder, a sensor might be forgotten about and left attached to the baby. However, she went on to say that this would be unlikely as the babies are checked regularly.

The size and weight increase of a wireless sensor attached to the skin was also identified as a potential problem as it could lead to pressure sores or interfere with phototherapy.

For a 23/24 weeker with very fragile skin, [I wonder] how much that bulk is going to impact on that baby; because obviously the leads are very light. So that would worry me, that they'd cause a pressure injury. [N3]

The concept of a wireless system was well received by all the participants, with many areas of care seen as potentially benefiting.

## Discussion

This study has built on the previous literature by seeking to understand the experiences that nurses and parents have with one particular aspect of the medical technology found in the NICU—the monitoring system, and especially its wires. Furthermore, we have explored how these stakeholders responded to the idea of a wireless system which might help to overcome some of the challenges they currently experience with the wired sensors.

Similar to previous studies in this field, we found that the wires interfere with parent–baby bonding despite the nurses' best efforts.^8^ The parents' ability to be involved in their babies' care has previously been found to be one of the most important factors in determining parental satisfaction with the care provided.^18^ Although babies would still be attached to the incubator by other life-supporting systems such as infusion lines and breathing support, the findings of this present study suggest that by removing the wires connecting the sensors to the vital sign monitors, we can improve the parent–baby bonding experience and therefore improve parent satisfaction. Furthermore, because the wires present a number of challenges to the provision of clinical care by the nursing team, moving to a wireless system may also reduce the discomfort suffered by the babies.

Previous work investigating nurses' attitudes towards technology in an intensive care setting found that, while it could complicate their everyday practice, it was recognised as being ‘good for patients’, bringing a range of benefits.^19^ In line with this, all participants interviewed in the present study had an overall positive attitude towards a wireless version of the monitoring system and many expressed the view that the wireless system would improve the kangaroo care experience. When concerns were raised, they centred on expectations that the size of the device must be small and unobtrusive, the data needs to be as good as the current system and the system needs to be completely reliable. While no adverse effects have been demonstrated to result from radio frequency radiation exposure within the regulated limits,^20^ it might plausibly have been raised as an issue given public concerns. However, none of the participants expressed concerns over this, indicating that it might not be a significant barrier to uptake.

Current standard clinical care requires many wires connecting multiple sensors and monitors to safely manage babies within the NICU. These wires pose additional challenges to the parents and nurses who work together to provide care for the babies. In particular, the wires can negatively impact the provision of kangaroo care and can complicate the repositioning and transportation of the babies. Developing a wireless system could mitigate these problems if the technology is implemented well and accepted by those in the NICU. Recent advances in wireless technology could realise this opportunity but there remain challenges in designing a device suitable for this unique environment.

To address these challenges, further work is planned to design and test a wireless vital sign monitoring system on the NICU. As part of the design work, user studies will be conducted which will further assess the impact the wireless system will have. By redesigning the interface between the sensor and the monitoring unit, we are influencing the relationship between various technologies and the babies, nurses, parents and others involved in their care. Prior work has shown that these relationships are complex,^8^ ^19^ and so we must be mindful of the potential for negative or unintended consequences. That said, this present study has shown that wireless monitoring on the NICU has great potential and that key user groups would welcome innovation in this area.
